# Flexible ureteroscopic lithotripsy with a suctioning ureteral access sheath for removing upper urinary calculi under local anesthesia

**DOI:** 10.3389/fsurg.2023.1242981

**Published:** 2023-08-31

**Authors:** Zhaolin Zhang, Song Leng, Tianpeng Xie, Yuanhu Yuan, Xiaoning Wang

**Affiliations:** ^1^ Department of Urology, First Affiliated Hospital of Gannan Medical University, Ganzhou, China; ^2^ First Clinical Medical College, Gannan Medical University, Ganzhou, China

**Keywords:** local anesthesia, ureteral access sheath, flexible ureteroscopic lithotripsy, urinary calculi, stone-free rate

## Abstract

**Objectives:**

We aimed to probe the safety and effectiveness of flexible ureteroscopic lithotripsy (FURL) with a suctioning ureteral access sheath (S-UAS) for removing upper urinary calculi under local anesthesia (LA).

**Materials and methods:**

The clinical data of 56 patients with upper urinary calculi treated by FURL with an S-UAS under LA during the period between September 2019 and November 2022 were analyzed retrospectively. For LA, intramuscular pethidine (1.0–2.0 mg/kg) and phenergan (25 mg) were administered 30 min prior to surgery, and oxybuprocaine hydrochloride gel was administered through the urethra at the start of the surgery. The S-UAS and flexible ureteroscope were used for FURL. Demographic characteristics, stone-related parameters, and clinical outcomes were analyzed.

**Result:**

A total of 66 procedures were performed successfully on 46 patients (Group A), who underwent unilateral surgeries, and on 10 patients (Group B) who underwent same-session bilateral surgeries. All 56 patients were operated upon without altering the anesthesia strategy, and none required additional analgesia. The mean stone sizes of the Group A and Group B patients were 20.24 ± 5.45 mm and 29.40 ± 3.89 mm, respectively. The mean operative times of the two groups were 53.04 ± 13.35 min and 90.00 ± 15.81 min, respectively. In Group A, the stone-free rates (SFRs) were 76.1% (35/46) and 85.1% (40/46) at postoperative day 1 and day 30, respectively. In Group B, the SFRs were 80.0% (16/20) and 85.0% (17/20), respectively. Four (8.7%) patients in Group A suffered complications such as fever, stent pain, urosepsis, and steinstrasse. In Group B, one (10%) patient suffered from fever.

**Conclusion:**

FURL, combined with an S-UAS under LA, is a feasible option and provides satisfactory clinical outcomes for appropriately selected patients.

## 1. Introduction

Urinary calculus is a common worldwide urological condition, and the prevalence rates vary among different regions, ranging from 1% to 13% (1). Currently, the major minimally invasive endoscopic surgical methods for urolithiasis are flexible ureteroscopic lithotripsy (FURL) and percutaneous nephrolithotomy (PCNL). FURL is recommended as a first-line option for renal calculi smaller than 20 mm (2). However, with the advent of a miniaturized flexible ureteroscope (FURS) and innovative technologies, it can also be applied for removing high-burden renal stones beyond 20 mm with good outcomes (3, 4).

FURL is regularly performed under general or regional anesthesia (5) but rarely under local anesthesia (LA) predominantly because of the pain caused by surgical procedures or ureteral damage caused by painful movement (6). However, for patients with absolute or relative contraindications to general or regional anesthesia, LA is a selective method. Only a few studies have reported the successful application of ureteroscopic lithotripsy under LA (6, 7), and all procedures reported in these studies were performed on the unilateral side. For bilateral upper urinary stones, simultaneous bilateral FURL has been reported as a favorable less-invasive alternative (8). In this study, we first present our experiences with FURL, combined with a suctioning ureteral access sheath (S-UAS) under LA, for removing unilateral or bilateral upper urinary calculi.

## 2. Materials and methods

### 2.1. Patients

The medical records of patients with upper urinary calculi who underwent FURL with an S-UAS under LA at the First Affiliated Hospital of Gannan Medical University during the period between September 2019 and November 2022 were retrospectively reviewed, and these patients were included in the study. The exclusion criteria were as follows: (a) lower urinary tract calculi; (b) middle or distal ureteral stones; (c) preoperative ureteral structure or calculous pyonephrosis; (d) combined with upper urinary carcinoma. For two patients, the method of treatment was changed to PCNL under LA because of a narrow ureter. Finally, a total of 56 patients were included in our study. All patients were diagnosed by preoperative urinary non-contrast computed tomography (NCCT). For patients with normal renal function, intravenous urography (IVU) was recommended. The stone size was defined as the largest diameter measured by NCCT, and for multiple stones or bilateral upper urinary stones, the size was the sum of the largest diameter of each stone. Urinalysis and urine culture were routinely examined and the stones were treated with appropriate antibiotics preoperatively. Preoperative demographic characteristics such as gender, age, American Society of Anesthesiologists (ASA) score, body mass index (BMI), surgical side, ipsilateral surgical history, midstream urine culture result, stone parameters, hydronephrosis, and preoperative ureteral stent placement were obtained according to medical records.

Ethical approval for the study protocol was obtained from the Ethics Committee of the First Affiliated Hospital of Gannan Medical University (proof number: 2023032706), and the study was performed in accordance with the Declaration of Helsinki (as revised in 2013). Written informed consent was obtained from all participants.

### 2.2. Surgical techniques

**Figure 1:**
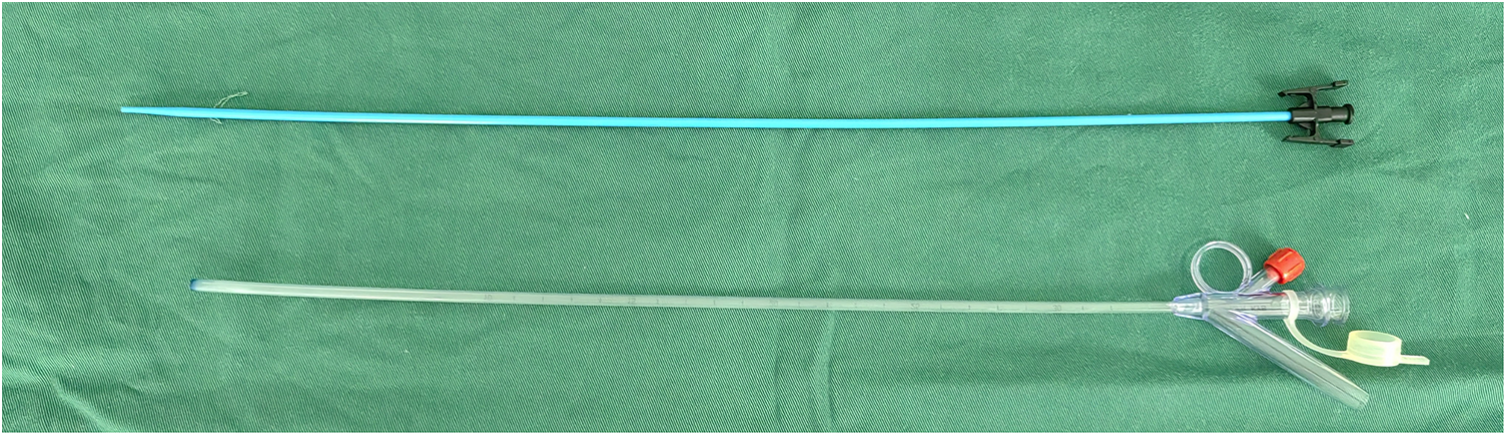
The suctioning ureteral access sheath.

**Figure 2:**
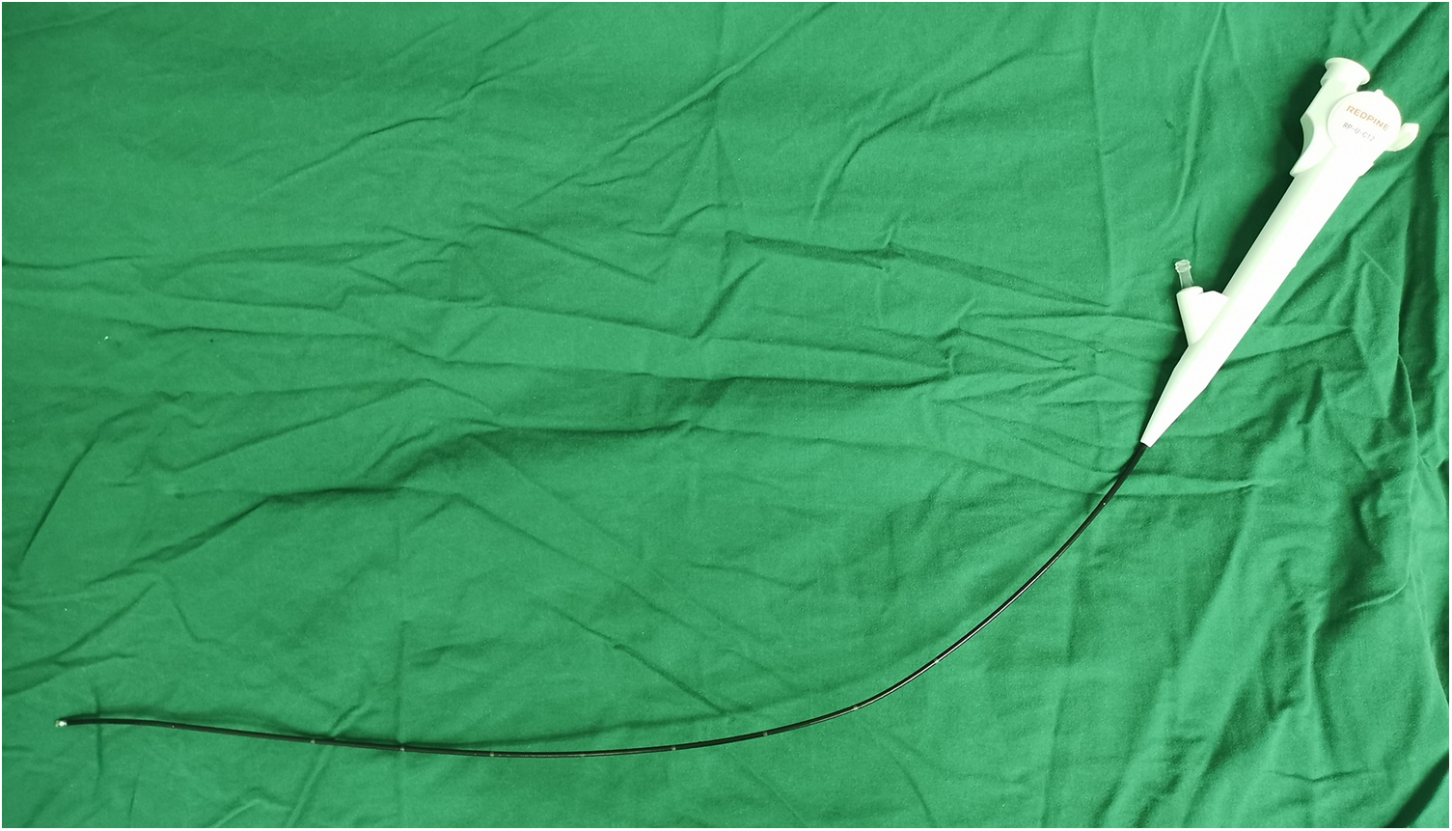
A single use flexible ureteroscope.

**Figure 3:**
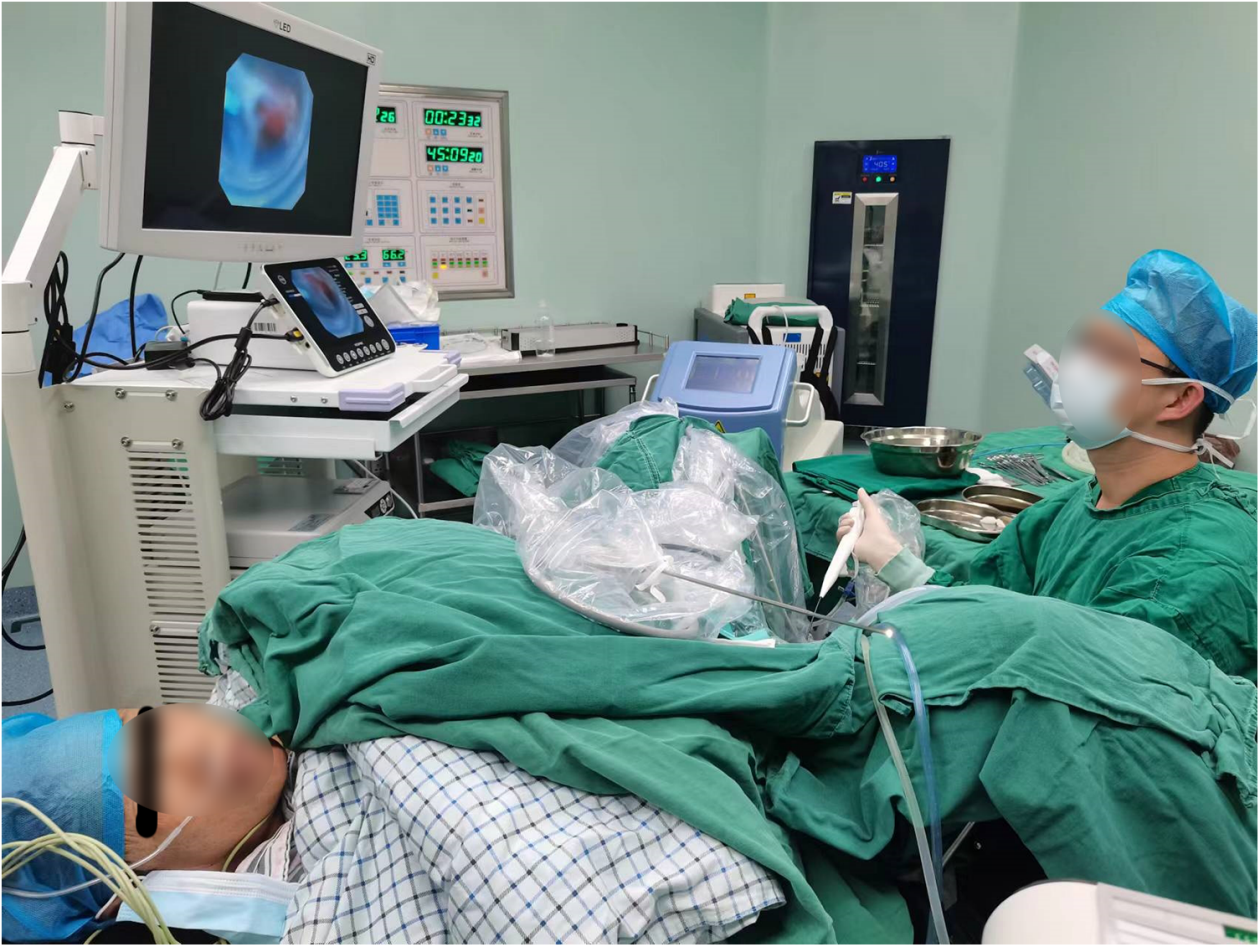
Patient was performed flexible ureteroscopic lithotripsy under local anesthesia.

All patients were explicated with all alternative therapeutic strategies and anesthetic methods. Written informed consent was granted before the operation. For patients who selected FURL under LA, intramuscular pethidine (1.0–2.0 mg/kg) and phenergan (25 mg) were administered 30 min prior to surgery. After the patients were placed in the lithotomy position, the oxybuprocaine hydrochloride gel (10 ml gel containing 30 mg oxybuprocaine) was injected into the urethra for mucosal anesthesia and lubrication. A ureteroscopy inspection was performed by using a semirigid 6/7.5 Fr ureteroscope, and then a guide wire was inserted in the ureter. If a proximal ureter stone was detected, the stone was pushed retrogradely to the renal pelvis. Under the guidance of the wire, an 11/13 F or 12/14 F S-UAS (Shenzhen Kang Yi Bo Technology Development Co., Ltd., Shenzhen, China), combined with a vacuum aspiration device, was inserted depending on the condition of the ureter (Figure 1), and the S-UAS was placed in the pyeloureteral junction. Then, a single-use FURS (Guangzhou Red Pine Medical Instrument Co., Ltd., Guangzhou, China) was used for inspection. The FURS had a wide deflecting angle that ranged upward at 275° and downward at 275°, the outer diameter was 8.7 F, and the working channel inner diameter was 3.6 F (Figure 2). After a comprehensive inspection of renal calices and stones, a 200 -μm laser fiber was inserted through the FURS, and a holmium:yttrium aluminum garnet (Ho:YAG) laser was applied to pulverize calculi by interchangeably setting different parameters. A low-energy setting (0.2–0.6 J) and a high range of frequency (20–30 Hz) was set for dusting, the fragmentation mode using higher energy ranged between 0.6 and 1.2 J, and the lower range of frequency was 5–20 Hz. A nitinol stone basket was applied to retrieve or relocate fragments when necessary. During the surgical procedure, the perfusion flow was set to 60–100 ml/min and the suctioning parameter of the vacuum device with negative pressure was set at −20 to −40 kPa. A part of the debris and dust was suctioned out through an S-UAS immediately. For some gravel particles, stone baskets or forceps were applied if necessary. After all renal stones were pulverized to the desired fragments and removed satisfactorily, a 5 F double-J stent was inserted routinely. Patients with bilateral upper urinary calculi underwent surgery in the same session, and the same surgeon operated on one side after completing another side. Figure 3 shows a patient who underwent FURL under LA. The procedures were performed by two expert surgeons, with each of them performing more than 250 FURL procedures per year.

The operative time, hemoglobin loss, visual analog scale (VAS) score, stone-free rate (SFR), and complication rate (CR) were analyzed. Kidney-ureter-bladder (KUB) graphy and/or urinary NCCT were performed at 1 day and 1 month after surgery, and a stone-free status was defined as “no remaining stone.” For patients who underwent bilateral FURL, the operation time was defined as “the total surgical time of two sides.” The double-J stent was routinely removed a month following surgery. For patients with ureteral stenosis or residual stones, a second procedure was performed 1 month after surgery.

## 3. Result

A total of 66 flexible ureteroscopic procedures were performed on 56 patients with upper urinary calculi, who included 46 patients (Group A) who underwent unilateral surgeries and 10 patients (Group B) who underwent same-session bilateral surgeries. In Group A, 22 patients underwent surgery on the left side and 24 patients on the right side. All procedures were successfully performed under local anesthesia without shifting to general or regional anesthesia, and none of them required additional analgesia during the performance of the surgeries.

Females constituted the majority of the two groupings. A total of nine patients in Group A and two patients in Group B were evaluated as high anesthesia risk (ASA III-V) patients. Comorbidities of the patients were hypertension, diabetes mellitus, coronary atherosclerotic heart disease, chronic obstructive pulmonary disease, renal insufficiency, and cerebral infarction. Two patients in Group A and one patient in Group B took daily aspirin, but there was no need to stop aspirin before FURL. The mean stone size was 20.24 ± 5.45 mm in Group A patients and 29.40 ± 3.89 mm in Group B patients. Preoperative ureteral stents on the surgical side were seen in 36 patients in Group A and 8 patients in Group B. The demographic characteristics and stone-related parameters are given in Table 1.

**Table 1:** Demographic characteristics and baseline data of unilateral and bilateral groups.

	Unilateral group	Bilateral group
Total number (*n*)	46	10
Age (years), mean ± SD	54.74 ± 12.50	52.80 ± 13.44
Gender, *n* (%)		
Male	5/46 (10.9%)	—
Female	41/46 (89.1%)	10/10 (100%)
BMI (kg/m^2^), mean ± SD	23.53 ± 3.48	24.07 ± 2.02
ASA score, *n* (%)		
I	6/46 (13.0%)	2/10 (20.0%)
II	31/46 (67.4%)	6/10 (60.0%)
III	9/46 (19.6%)	2/10 (20.0%)
Comorbidities, *n* (%)		
Hypertension	7/46 (15.2%)	3/10 (30.0%)
Diabetes mellitus	10/46 (21.7%)	2/10 (20.0%)
Coronary atherosclerotic heart disease	2/46 (4.3%)	1/10 (10.0%)
Chronic obstructive pulmonary disease	3/46 (6.5%)	1/10 (10.0%)
Renal insufficiency	11/46 (23.9%)	4/10 (40.0%)
Cerebral infarction	1/46 (2.2%)	—
Operative side, *n* (%)		
Left	22/46 (47.8%)	
Right	24/46 (52.2%)	
Bilateral		10/10 (100%)
History of surgery on the surgical ipsilateral side, *n* (%)		
ESWL	2/46 (4.3%)	—
RIRS	5/46 (10.9%)	3/20 (15.0%)
PCNL	2/46 (4.3%)	4/20 (20.0%)
Laparoscopic surgery	2/46 (4.3%)	1/20 (5.0%)
Midstream urine culture, *n* (%)		
Positive	17/46 (37.0%)	3/10 (30.0%)
Negative	29/46 (63.0%)	7/10 (70.0%)
Stone size (mm), mean ± SD	20.24 ± 5.45	29.40 ± 3.89
Stone hardness (HU), mean ± SD	835.46 ± 318.28	819.6 ± 220.09
Stone location of the surgical ipsilateral side, *n* (%)		
Pelvis	10/46 (21.7%)	6/20 (30.0%)
Upper calyx	3/46 (6.5%)	1/20 (5.0%)
Middle calyx	4/46 (8.7%)	3/20 (15.0%)
Lower calyx	7/46 (15.2%)	2/20 (10.0%)
Proximal ureter	8/46 (17.4%)	2/20 (10.0%)
Multiple location	14/46 (30.4%)	6/20 (30.0%)
Hydronephrosis at the surgical ipsilateral side, *n* (%)		
No	11/46 (23.9%)	5/20 (25.0%)
Mild	20/46 (43.5%)	5/20 (25.0%)
Moderate	12/46 (26.1%)	7/20 (35.0%)
Gross	3/46 (6.5%)	3/20 (15.0%)
Preoperative ureteral stent existence at the surgical side, *n* (%)		
Yes	36/46 (78.3%)	16/20 (80.0%)
No	10/46 (21.7%)	4/20 (20.0%)

ESWL, extracorporeal shock wave lithotripsy; RIRS, retrograde intrarenal surgery; HU, Hounsfield unit.

A total of 44 patients were prestented, including four patients with high general anesthesia risk who underwent regular replacement of a double-J stent because of calculous hydronephrosis, three patients who received a stent for sepsis; the remaining 37 patients were prestented for ureter dilation. In Group A, all prestented patients and two patients without preoperative stent were inserted with 12/14 F S-UAS, and the remaining patients were inserted with 11/13 F S-UAS. All 10 patients in Group B were treated with 12/14 F S-UAS. The mean operative time was 53.04 ± 13.35 min in Group A patients and 90.00 ± 15.81 min in Group B patients, and the mean hemoglobin loss was −4.78 ± 9.22 g/L and −4.10 ± 9.09 g/L, respectively. The intraoperative mean visual analog scale scores and scores at 6 and 24 h after surgery in Group A patients were 3.83 ± 0.53, 2.23 ± 0.64, and 1.22 ± 0.79, respectively. In Group B patients, the intraoperative mean visual analog scale scores and scores at 6 and 24 h after surgery were 3.10 ± 0.74, 2.20 ± 0.63, and 1.20 ± 0.63, respectively. The mean postoperative hospitalization time was 3.43 ± 1.70 days and 3.20 ± 1.40 days, and 35.7% (20/56) of patients had a postoperative hospitalization time of more than 3 days, predominantly because of the intrinsic features of our medical strategy and insurance policy and the tertiary hospital referral system and not because of surgery.

In our study, all patients received postoperative KUB. For patients with suspicious fragments based on KUB, a CT was done, and 30.3% (17/56) of patients were checked with NCCT. The SFRs of Group A patients at postoperative day 1 and day 30 were 76.1% and 85.1%, respectively. In Group B patients, the SFRs of the surgical ipsilateral side at postoperative day 1 and day 30 were 80.0% and 85.0%, respectively.

Four (8.7%) patients in Group A suffered from the following complications: 1 (2.2%) patient suffered from fever (Clavien grade I), 1 (2.2%) patient who had stent pain (Clavien grade I) was treated with a steroidal anti-inflammatory agent, 1 (2.2%) patient who had urosepsis only needed additional antibiotics (Clavien grade II), and 1 (2.2%) patient with steinstrasse was treated with ureteroscopic lithotripsy under LA. In Group B, 1 (10%) patient suffered from fever (Clavien grade I). No ureter injuries were observed in the two groups. More details of the clinical outcomes are given in Table 2.

**Table 2:** Clinical outcomes of unilateral and bilateral groups.

	Unilateral group	Bilateral group
Total number, (*n*)	46	10
Operative time (min), mean ± SD	53.04 ± 13.35	90.00 ± 15.81
Hemoglobin loss (g/L), mean ± SD	−4.78 ± 9.22	−4.10 ± 9.09
Postoperative hospitalization (days), mean ± SD	3.43 ± 1.70	3.20 ± 1.40
SFR of the surgical ipsilateral side at postoperative day 1, *n* (%)	35/46 (76.1%)	16/20 (80.0%)
SFR of the surgical ipsilateral side at postoperative day 30, *n* (%)	40/46 (85.1%)	17/20 (85.0%)
Intraoperative VAS score	3.83 ± 0.53	3.10 ± 0.74
VAS score at 6 h postoperatively	2.23 ± 0.64	2.20 ± 0.63
VAS score at 24 h postoperatively	1.22 ± 0.79	1.20 ± 0.63
Total complications, Clavien grade classification, *n* (%)	4/46 (8.7%)	1/10 (10.0%)
Fever (>38°C) (G I)	1/46 (2.2%)	1/10 (10.0%)
Stent pain (G I)	1/46 (2.2%)	—
Urosepsis only needing additional antibiotics (G II)	1/46 (2.2%)	—
Steinstrasse (G III)	1/46 (2.2%)	—

G, grade.

## 4. Discussion

Because of the miniaturization of novel FURS and the development of lithotripsy devices, FURL is being increasingly performed nowadays because of its superior minimally invasive characteristics and satisfactory success rates. FURL is usually performed under general or regional anesthesia by anesthesiologists and urologists (5) but is rarely performed under LA. As the population is aging rapidly in China (9), the proportion of older patients with urolithiasis shows an increasing trend (10). Elderly people are associated with age-related functional decline of organ systems, decreased physiological reserve (11), and non-communicable diseases or comorbidities (9), which result in high anesthetic risk. For these patients with high-risk anesthesia, LA is a feasible option (7).

We first reported FURL, combined with S-UAS, for urinary calculi under LA. Unlike the LA method described in a previous study conducted by Pai et al. (7), which used only a lubricating gel per urethra, our LA procedures included two steps: intramuscular pethidine and phenergan were used half an hour prior to surgery for analgesia and sedation, and oxybuprocaine hydrochloride gel was infused into the urethra at the start of the surgery. Preoperative use of pethidine and phenergan could increase patient tolerance for surgical operations because of their analgesic and sedative effects (12). No patient in our study abandoned surgery because of pain or ureteral injury. Moreover, a second ureteroscopic lithotripsy under LA for a patient with postoperative steinstrasse was also successfully completed.

For patients who underwent LA, preoperative ureteral stenting was advised, and these patients constituted 78.3% in Group A and 80.0% in Group B. The benefits of prestenting were continuous relief of hydronephrosis and alleviation of obstructive pain caused by edematous mucosa (13). Stent placement dilates the ureter, facilitates insertion of the ureteral access sheath (UAS) (14), and improves the initial success rate (15). Although all patients in our study were successfully inserted an S-UAS, prestented patients were inserted large-caliber UASs.

In our study, we evaluated the intraoperative and postoperative VAS scores of patients who underwent FURL under LA. All procedures were successfully completed without additional analgesia, and the mean intraoperative VAS scores were 3.83 ± 0.53 in Group A and 3.10 ± 0.74 in Group B. A previous study indicated that high pelvis pressure was associated with intraoperative pain (16, 17). According to the working theory of suctioning design, the application of the S-UAS in our study can help maintain low intrarenal pressure (18), which can reduce intraoperative pain and decrease complications associated with high intrarenal pressure.

Except for one patient who needed an oral steroidal anti-inflammatory agent after surgery because of stent pain, all patients tolerated postoperative pain. Multiple studies have focused on factors related to postoperative pain after FURL (13, 19–22). Oğuz et al. observed that female patients, a large stone diameter, high residual fragments, and a prolonged dwell time of UAS in the ureter were main factors associated with postoperative pain in patients who underwent FURL (19). Tighe et al. reviewed 333,000 pain scores following surgery and detected that female patients experienced higher pain scores (20). Mustafa reported that ureteral stenting was associated with postoperative discomfort (13). In contrast, another study demonstrated that double-J stent placement might lessen postoperative discomfort or relieve loin pain after FURL (13, 21). Postoperative catheter indwelling could also increase postoperative VAS scores (22). Although risk factors such as being female, large stone size, and routine double-J stent placement were observed in our study, the mean postoperative VAS score was low. The reasons listed below can help explain our results. First, stone particles can be immediately suctioned out through the S-UAS, which can shorten the dwell time of the UAS and the total surgical time. Second, the application of S-UAS can drain renal fluid in time to maintain low renal pressure, even at high irrigation flow, which can help maintain clear surgical vision, improve the efficiency of the lithotripsy procedure, and reduce operation time. Third, a postoperative catheter was avoided in all patients under LA.

The SFRs at postoperative day 30 were 85.1% (40/46) in Group A patients and 85.0% (17/20) in Group B patients, which were consistent with the outcomes of the study conducted by Pai et al. (7). However, the median stone size in Pai et al.'s study was 8 mm, which was considerably smaller than that in our study (20.24 ± 5.45 mm). In addition, only 64.7% (55/85) patients in Pai et al.'s study had renal or proximal ureteral stones and underwent FURL (7). To our knowledge, except for our study and Pai et al.'s study, no research with regard to FURL under LA was seen in the PubMed database. Park et al. reported that the SFR was 83% (5/6) for patients with upper ureteral calculi, but all underwent ureteroscopic lithotripsy under LA. Compared with the SFR (88.8%) of FURL, combined with S-UAS under general anesthesia (23), our SFR was comparable.

The overall CR in our study was 8.7% in the unilateral group, which was comparable with outcomes reported in a previous study (7). No ureteral injury caused by painful movement was observed in the two studies. Infectious CR (fever, urosepsis, and septic shock) was 4.4% (2/46) in the unilateral group, which was comparable with a study in which an S-UAS was used under general anesthesia (23), but it was lower than in a study in which the traditional UAS was used (23). This difference was mainly due to the status of low intrarenal pressure maintained by the application of the S-UAS. Moreover, infectious substances can be suctioned in time, and the amount of infectious sources for pyelovenous backflow is reduced.

Same-session bilateral FURL is a favorable therapy with a satisfactory SFR and an acceptable CR; however, it may prolong operation time and cause renal damage (8). Ten patients underwent same-session bilateral FURL in our study, and the SFR (85%) and postoperative CR (10%) were similar to the outcomes of a previous study (8). These patients were strictly selected, and the total stone size of each patient was less than or close to 30 mm.

Our study had several limitations. A major limitation was that it was a retrospective study with a limited sample size, and therefore, potential patient selection bias could not be ruled out. Second, a control group was lacking in the study. Third, we recorded the intraoperative VAS score, but the VAS score of different surgical procedures were not recorded, these procedures included ureteroscopy inspection, UAS insertion, ureteroscopic manipulation, and lithotripsy procedures. Therefore, a professionally designed study with a large sample size is recommended for the future.

## 5. Conclusion

FURL combined with an S-UAS under LA is a feasible option and provides satisfactory clinical outcomes for appropriately selected patients. A professionally designed study with a large sample size is recommended for the future.

## Data Availability

The original contributions presented in the study are included in the article/Supplementary Material, further inquiries can be directed to the corresponding author.
